# Modified polyether-sulfone membrane: a mini review

**DOI:** 10.1080/15685551.2017.1398208

**Published:** 2017-11-02

**Authors:** Noof A. Alenazi, Mahmoud A. Hussein, Khalid A. Alamry, Abdullah M. Asiri

**Affiliations:** ^a^ Faculty of Science, Department of Chemistry, King Abdulaziz University, Jeddah, Saudi Arabia; ^b^ Polymer Chemistry Lab., Faculty of Science, Chemistry Department, Assiut University, Assiut, Egypt; ^c^ Center of Excellence for Advanced Materials Research (CEAMR), King Abdulaziz University, Jeddah, Saudi Arabia

**Keywords:** Polyether-sulfone, membrane, chemical modifications, amination process, surface modification, coating

## Abstract

Polyethersulfone has been widely used as a promising material in medical applications and waste-treatment membranes since it provides excellent mechanical and thermal properties. Hydrophobicity of polyethersulfone is considered one main disadvantage of using this material because hydrophobic surface causes biofouling effects to the membrane which is always thought to be a serious limitation to the use of polyethersulfone in membrane technology. Chemical modification to the material is a promising solution to this problem. More specifically surface modification is an excellent technique to introduce hydrophilic properties and functional groups to the polyethersulfone membrane surface. This review covers chemical modifications of the polyethersulfone and covers different methods used to enhance the hydrophilicity of polyethersulfone membrane. In particular, the addition of amino functional groups to polyethersulfone is used as a fundamental method either to introduce hydrophilic properties or introduce nanomaterials to the surface of polyethersulfone membrane. This work reviews also previous research reports explored the use of amino functionalized polyethersulfone with different nanomaterials to induce biological activity and reduce fouling effects of the fabricated membrane.

## 1. Introduction

**Figure 1.**
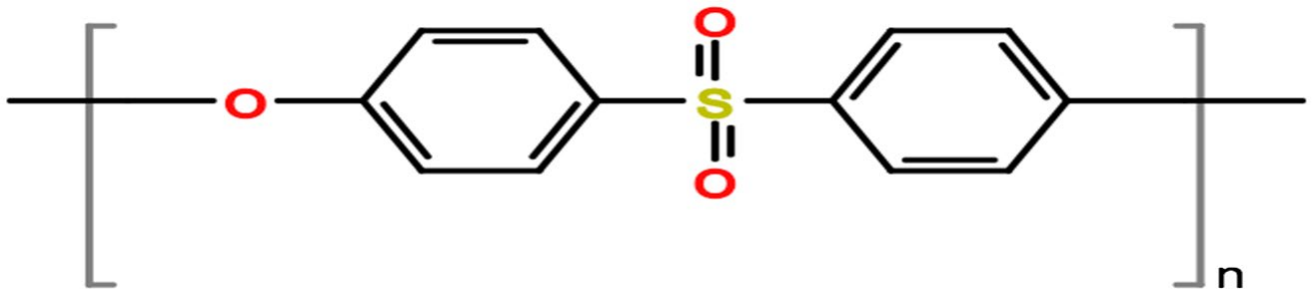
The chemical structure of polyethersulfone (PES).

Polyethersulfone (PES), as shown in Figure 1, is considered one of the well-known polymeric material to be used to make ultrafiltration (UF), microfiltration (MF), and gas separation membranes since it provides unique properties such as outstanding thermal, mechanical, hydrolytic strength in both hot and wet environments [1,2]. PES is chemically made from bisphenol A and dichlorodiphenylsulfone through a condensation reaction, and it proceeds through aromatic nucleophilic replacement mechanism [3,4]. PES membrane is transparent and amorphous in the structure and has a high *T*_*g*_ up to 225 °C [5,6]. There are different manufactures produce PES commercially such as BASF company with trade names as Ultrason E and Ultrason S, Solvay company with a trade name as Radel PES, and Sumitomo chemical company with a trade name as Sumika excel. The most used products to synthesize PES membrane in research studies are Ultrason product with molecular weight of 58 kDa and Radel product with molecular weight of 15 kDa [7].

Although PES is a very critical material used in membrane technology for different applications, the hydrophobic properties of PES limits its applications. Research reports have found that hydrophobicity is one main reason for membrane biofouling. Biofouling is alluded to as the undesirable accumulation and growth of biofilms. A biofilm is a deposition of microbial cells in which these cells adhere to the surface of the membrane (not expelled by delicate washing) and covered in a lattice of extracellular polymeric substances (EPS). When PES membrane is used in water purification, microorganisms which present in almost all aquatic environment, convert soluble salts and nutrients present in water to materials that semisolids or solids resulting in blocking the membranes [8,9]. Consequently, problems related to membrane quality start to appear such as increasing the cost of membrane maintenance, decreasing the lifetime of membranes as well as poor separation performance [9]. Chemical modification of PES membrane is used as a fundamental solution to increase the hydrophilicity and to develop anti-fouling membrane materials [10,11].

Using hydrophilic membrane is not always a valuable solution to avoid membrane-fouling effects since this type of membrane has possibility to swell in water and provide less mechanical as well as less thermal strength. An alternative solution to prevent fouling is to introduce hydrophilic functional groups into a hydrophobic polymer with unique mechanical properties such as PES. This chemical modification allows the chemical modified PES to combine properties between hydrophobicity characteristics such as excellent mechanical and thermal properties and hydrophilic characteristics to avoid fouling and increase the permeate flux [12]. It is important to highlight that one of the fundamental objective in membrane technology is to design membranes, which provide a highest permeate flux with a highest solute rejection while keeping the costs of the membrane production in minimum [13]. Introduction of hydrophilic functional groups can be done either by copolymerization of hydrophilic monomers with hydrophobic monomers to produce new polymer materials or by using a hydrophobic polymer as the main polymer material and hydrophilic groups introduced on the surface of this polymer. Hydrophilic functional groups that can be introduced onto PES are usually sulfone, carboxyl, hydroxyl, and amine functions [12].

Modification of PES membranes is based onto three methodologies, which are surface treatment including physical adsorption, UV irradiation, plasma treatment, etc., blending method, which is considered as a surface treatment, and bulk modification [2,12].

This work covers the novel chemical modification of PES membrane, which they have been previously explored [2,8,14]. In addition, this review aims to explore briefly recent studies that introduced amine functions to PES membrane either to increase the hydrophilicity of the polymeric membrane or to introduce nanomaterials/monomers/enzymes on the surface of PES membrane. As far as we know, there are no review papers covered the aminated-PES (NH_2_-PES) and their utility with nanomaterials/monomers/enzymes.

## 2. Modification of PES membranes

### 2.1. Surface modification

Surface modification of the PES membranes has been used as an attractive methodology to change the surface of the hydrophobic PES membranes so that hydrophilic properties are introduced on the surface while keeping the membrane backbone unmodified. Hydrophilic properties of the modified membrane prevent organic hydrophobic compounds from accumulation and attachment on the PES membrane surface. The hydrophilicity of PES membrane is measured by the contact angle which is in turn affected by different factors such as the membrane roughness, the porosity, the pore size, and the pore size distribution. When the value of the pore size of the membrane is high, a low value is obtained for the contact angle which means that the membrane is hydrophilic. However, when the roughness of the membrane surface rises up, it means the contact angle increases, which affects the hydrophilicity measurements measured by the contact angle [8]. There are different techniques used to enhance the hydrophilicity of the PES membrane through surface modification including coating techniques, blending method, plasma treatment, UV grafting, and surface initiated atom transfer radical polymerization (SI-ATRP) which have been used to attach hydrophilic functional groups on PES membrane surface [2,8,7].

#### 2.1.1. Coating techniques

**Figure 2.**
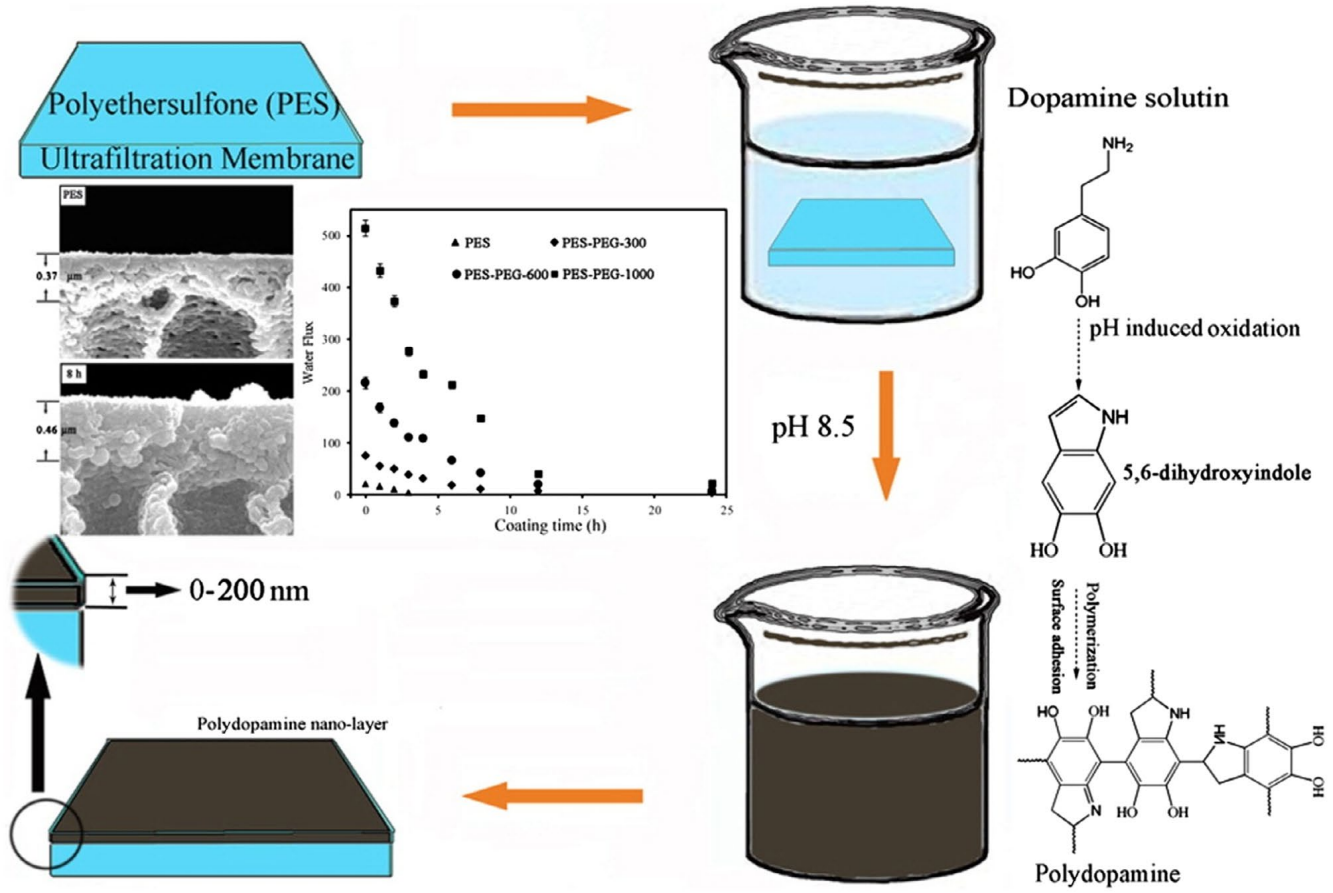
An illustration of the DA polymerization induced by pH and adhesion on the membrane of PES [22].

Coating techniques can be done by different methods including physical adsorption by non-covalent bonds [15]. The physical adsorption method is involved the attachment of thin hydrophilic layer on the surface of the membrane in order to increase the hydrophilicity of the membrane [12,16]. Adsorption occurs through electrostatic interactions, hydrophobic interactions, hydrogen interactions, and chemical reactions with functional groups that are present on the membrane surface [12,17]. Coating techniques can be done also through coating with a monolayer using Langmuir–Blodgett or analogous methods [15,18], deposition from a glow discharge plasma [15,19], and casting between two polymer solutions by simultaneous spinning such as a triple orifice spinneret [15,20,21]. There are several research studies have used coating techniques to modify the surface of PES membrane. For instance, Cheng et al*.* [22] synthesized PES membrane coated with poly dopamine (DPA) in alkaline solution of dopamine (DA) as shown in Figure 2, DA is oxidized at alkaline pH then polymerized to form a cross-linked poly dopamine (PDA) arrangement which could adhere onto many solid materials. The results of the study showed that both the hydrophilicity and blood compatibility of the coated PES membrane with PDA enhanced significantly.

Another a study by Reddy et al. [16], PES membrane is modified with poly (sodium 4-styrene sulfonate) (PSS) by permeation of PSS solution through PES membranes during 100 min. Preadsorption of PSS can take place on the surface and inside the pores. This can be determined based on molar mass of PPS polymer in contrast with the membrane pore size. The results of this study showed that the surface modified PES membranes with PSS have a better antifouling effects than the unmodified membrane. The coating techniques can be used easily, and it is a way to efficiently improve the PES membrane without disturbing the mechanical property of PES membranes. However, one main drawback of coating techniques is that the layer of hydrophilic polymers and monomers can be degraded with membrane long usage. Also, this method is not environmentally friendly since it involves the use of chemicals under risky and hazardous conditions in order to attach the hydrophilic polymer on the surface [8].

#### 2.1.2. Grafting covalent bonds by plasma and UV irradiation

Plasma treatment, which is produced by ionization of a gas or water, is a technique used to modify PES membrane and to generate radicals acting as active sites for graft polymerization [12,23]. Gas Ionization process occurs through electrical discharge parameters at elevated frequencies by microwaves and radio wave frequency. The hydrophilicity of the membrane surface is increased through the active species that are produced in the plasma keeping the backbone of the polymer unmodified. Depending on the plasma means, this technique provides new functional groups on the membranes surface with new characteristics. There are several gases have been used in plasma treatment to generate plasma species such as CF_4_, Ar, O_2_, H_2_, He, Ne, N_2_, CO_2_, and H_2_O. These ionized plasma species attack the surface of the membrane to create radical sites. The generated radicals attack specific bonds such as C–C, C–H, and C–S bonds except the aromatic C–H and C–H bonds. The produced radicals then react with gas species, and the residual radicals react with O_2_ and N present in the atmosphere. When CO_2_ plasma technique is used, oxygen species introduced on the membrane surface in types of acid, ester, and carbonyl functional groups. H_2_O plasma method produces hydroxyl, carboxyl, and carbonyl functional groups whereas plasma treatment species containing nitrogen functional groups produce amide, amine, and imine functional sites on the surface, which eventually increase the hydrophilic properties of the membrane. Subsequently, the plasma technique provides membranes with better antifouling effects, but further plasma modification could possibly cause a degradation in the membrane. Also, chain migration causes slow damage to the surface which causes eventually what is called hydrophobic recovery. FTIR and XPS techniques are used to characterize the functional groups that are induced by plasma treatment. Although this technique is effective, it has drawbacks such as the time dependency of the induced changes [2,12].

**Figure 3.**
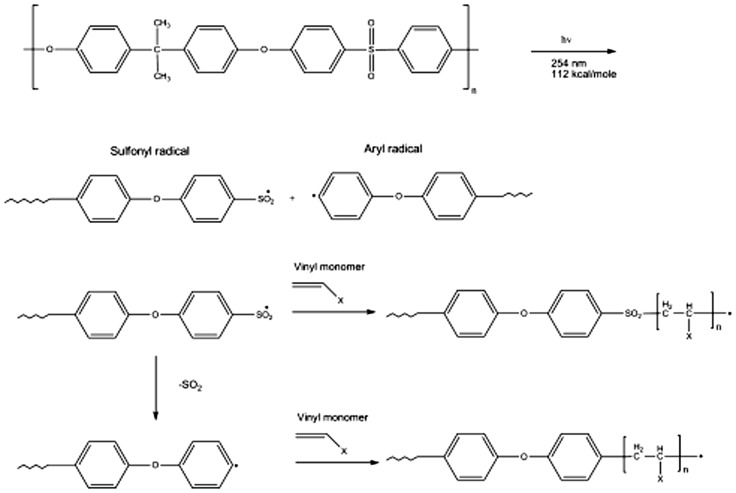
UV-induced grafting Mechanisms for PES membranes [12].

UV grafting technique is used because of simple and un-expansive. PES membrane can be easily functionalized under UV irradiation without the presence of photo-initiators since the sulfonyl groups in the polymer chain are sensitive to UV lights which creates radical sites for polymerization [24]. However, a suitable selection of wavelength is a critical factor in this method. The UV grafting mechanism is first started by light absorption by the phenoxy-phenyl chromophores present in the PES backbone. A homolytic cleavage occurs as a result of the photoexcitation of a C-S bond at the sulfonic group sites in the polymer chain which causes a splitting in the polymer backbone as shown in Figure 3, yielding two radicals which are aryl radical and sulfonyl radical. These resulted radicals are reactive species that start polymerization beside there is a possibility that sulfonyl radicals could lose it sulfonyl group to produce another aryl radical. By using the immersion technique, UV irradiation is used in the presence of water or methanol. In this method, the membrane is immersed in a solution containing monomers such as vinyl monomers. UV irradiation creates radical sites which react with monomers. Then, the monomers are covalently bond to the membrane through polymerization that induce by the free radicals. Afterwards, the reaming or unreactive species are removed by washing them with deionized water [2,12]. Although plasma and UV irradiation methods are considered very promising and effective technique in small-scale applications in chemistry laboratory, it is not practical for industrial large-scale applications [2,25].

#### 2.1.3. Other surface modification methods

There are techniques such as thermal induced polymerization and grafting functional groups which is an approach to modify the surface of PES membrane. In this method, chemical initiators or cleavage agents are always used. This technique is effective and useful to immobilize biomolecules compounds such as enzymes, protein, and amino acid which could covalently attached on the surface of PES membrane by chemical reaction [2,26].

Atom transfer radical polymerization (ATRP) is relatively new technique to graft polymer, and it is considered easy and efficient since it requires reasonably mild conditions and allows different vinyl monomers to be polymerized in a controlled way with well-defined structure. There are three grafting techniques used surface-initiated ATRP so far to modify PES membrane which are grafting-through, grafting-to, and grafting-from. However, there are a few research studies covered the surface-initiated ATRP method used for PES membrane modification [2,27].

### 2.2. Blending technique

Blending of hydrophilic functional groups with PES membrane is considered one of the easiest strategy to enhance the hydrophilicity of the membrane surface. Usually, the blending technique changes the property of the PES membrane since new materials are blended with PES. In addition, the polymer blending can be categorized as homogenous or heterogynous [28,29]. Polymeric blending allows hydrophobic PES membrane which exhibited high chemical and mechanical strength to be blended with hydrophilic polymeric materials such as polyethylene glycol (PEG), polyvinylpyrrolidone (PVP), or (polyethylene oxide-b-polypropylene oxide-b-polyethylene oxide [30]. PES membrane can be also blended with the inorganic nanomaterials such as silica, silver, aluminum oxide and titanium dioxide [7,31]. In copolymer blending, for instance, Wang et al. [32] blended PES membrane with the copolymers N,N-dimethyl-N-methacryloxyethyl-N-(3-sulfopropyl) (DMMSA)-butyl methacrylate (BMA) for bovine serum albumin (BSA) separation. In this work, the hydrophilic (DMMSA) polymer reacted with hydrophobic (BMA) polymer to produce (DMMSA-BMA) copolymer. Then, DMMSA-BMA materials blended with PES membrane to produce a new antifouling ultrafiltration membrane. The results of the study showed the hydrophilic functional groups favorably concentrated at the membrane surface, and the antifouling of the modified membrane enhanced in comparison with unmodified membrane. In using inorganic nanomaterials blending, for example, Sotto et al. [33] modified PES with titanium dioxide (TiO_2_) nanomaterials to produce PES–TiO_2_ membranes which was synthesized form the casting solution that consist of various compositions of polymer solvents (DMF and EtOH) and TiO_2_ additive. In this work, the results exhibited that the membrane permeate flux and solute rejection rate are depended on the concentrations of both TiO_2_ and EtOH, and the structure of the PES–TiO_2_ membranes has changed from a sponge-like to a finger-like structure after the modification. The antifouling of the modified PES TiO_2_ membrane enhanced significantly. However, although this technique is considered the most convenient and easy method, there are some drawbacks such as the inevitable leaching of the blended hydrophilic materials from the polymeric membranes after long-term use [2,33].

### 2.3. Bulk modification

Bulk modification is when a functional group is introduced to the backbone of the polymeric chain such as sulfone (–SO_3_H), hydroxyl (–OH), carboxyl (–COOH), amino (–NH_2_), or fluoro benzene, or a combination of these groups are introduced in order to improve the hydrophilicity of PES.

#### 2.3.1. Sulfonation (–SO_3_H)

**Figure 4.**
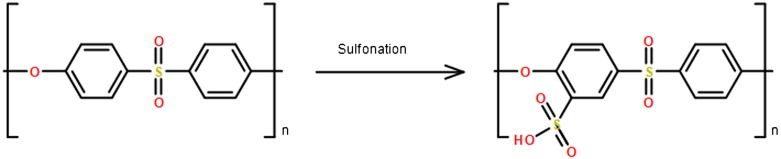
Sulfonation of PES.

PES chemical modification has been applied in order to reduce the hydrophobicity of PES membrane. Sulfonation reaction is one of the chemical modification methods used to introduce negatively charged sulphonic acid groups to the PES membrane as shown in Figure 4. There are two types of sulfonation that can be used to introduce SO_3_H groups to the polymeric chain; it can be done by a heterogeneous treatment using sulfonating agents or a homogenous treatment using pre-sulfonating monomers in the polymerization reaction [34,35]. The heterogeneous treatment is considered more popular than the homogenous treatment due to the low cost and straightforwardness [36]. The sulfonation of PES occurs via electrophilic aromatic substitution reaction in which a hydrogen atom in the aromatic polymeric chain is replaced by a sulfonic group, and it is localized at the ortho position in relation to the ether linkage that is presented in the PES chain. It is well known that sulfonation, electrophilic reaction, is enhanced by electron donating groups whereas electron withdrawing groups exhibit the opposite effects. Thus, sulfonation of PES is challenging due to the withdrawing effects of electrons presented in sulfonic groups causing the aromatic chain to be deactivated for substitution. Sulfonation of PES has been reported previously [37,42–52], and the synthesis is done before membrane formation using different sulfonating agents such as chlorosulfonic acid (ClSO_3_H) [37–42] oleum (SO_3_ in H_2_SO_4_) [43], sulfur trioxide-triethylphosphate complex (SO_3_-TEP) [44,45], sulfur trioxide (SO_3_) [43,46], trimethyl silylchlorosulfate ((CH_3_)_3_SiSO_3_Cl) [46–50], sulfuric acid (H_2_SO_4_) [51], sulfuric acid (H_2_SO_4_) as a solvent followed by chlorosulfonic acid a sulfonating agent (ClSO_3_H) [52–55]. The sulfonation is identified by the degree of sulfonation (DS) which depends on four factors; the reaction time, sulfonating agents’ molar ratio, reaction temperature, and polymer. When the reaction time is long, the sulfonation rate increases until it slows down while the temperature increases DS, but it could contribute to polymer chain degradation. DS is can be measured by ion exchange capacity, proton nuclear magnetic resonance (^1^H NMR), or infrared radiation (IR) spectroscopy [12].

#### 2.3.2. Carboxylation (–COOH)

**Figure 5.**
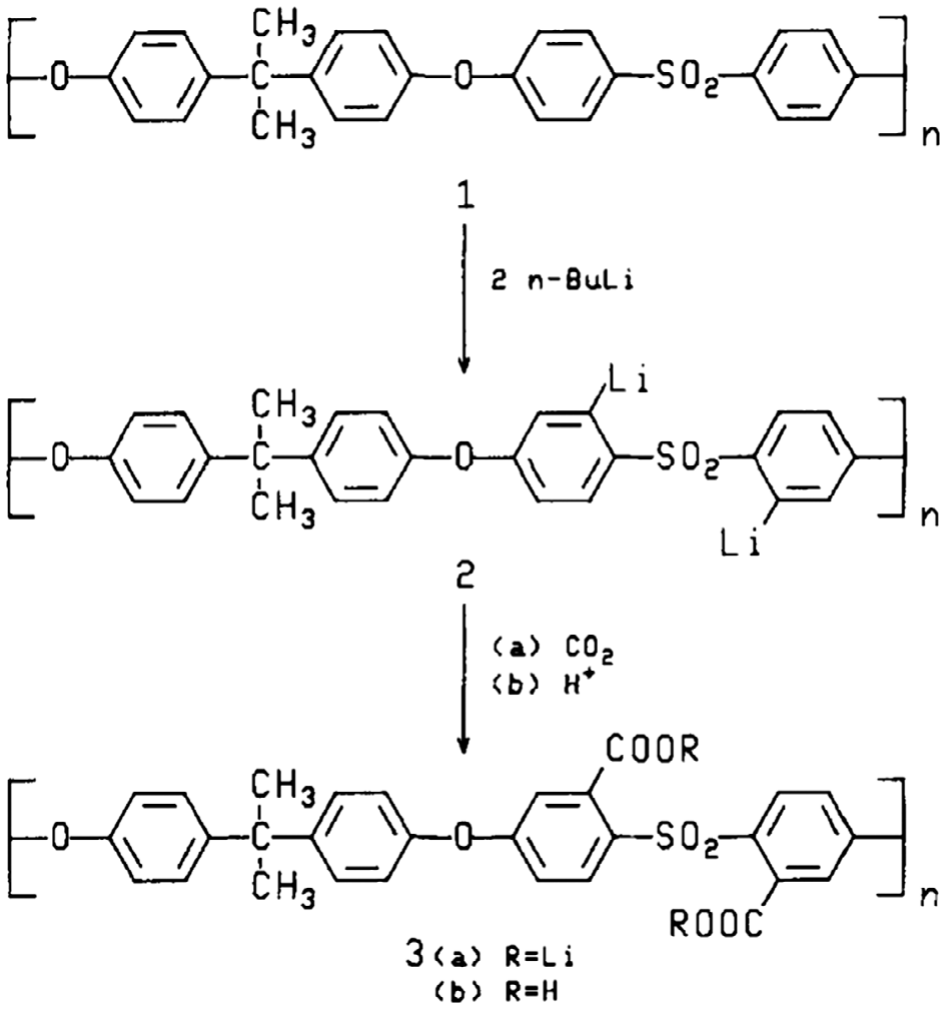
Two step process for the carboxylation of polysulfones [56].

Carboxylic groups are introduced to PES backbone in a similar way to sulfonation in which a carboxylic group replaces a hydrogen atom at the aromatic PES in an aromatic substitution reaction. Carboxylation of PES has been shown to improve the hydrophilicity of PES and Polysulfones in a similar way to the sulfonation of PES. Carboxylation of PES is not considered difficult, and it is done by two steps process, lithation followed by carboxylation. When PES is lithiated, two Li atoms are generated at the ortho position relative to the ether linkage per repeat unit due to the activating and directing effects of sulfonic groups in the aromatic chain towards lithation. Next, the lithiated species in the polymeric chain are reactive towards electrophilic substituents, and in this case is carboxylic functions [12,56]. Guvir et al. [56] has prepared carboxylated polysulfones as shown in Figure 5 with different degree of substitution (DS) between 0.29 and 1.90 acid groups per repeat unit. Different ratios of n-butyllithium was used to lithiate polysulfones in THF at a low temperature up to – 50 °C, and this low temperature was used in order prevent crosslinking reaction from occurring in the polymer. Then, when the lithiation of the polymer was completed, dry ice (CO_2_) was added to the reaction mixture which resulted in the formation of lithiated carboxylate derivative of the polymer. The carboxylate derivatives were then converted to carboxylic functions (–COOH) by acidifying them using hydrochloric acid [56].

**Figure 6.**
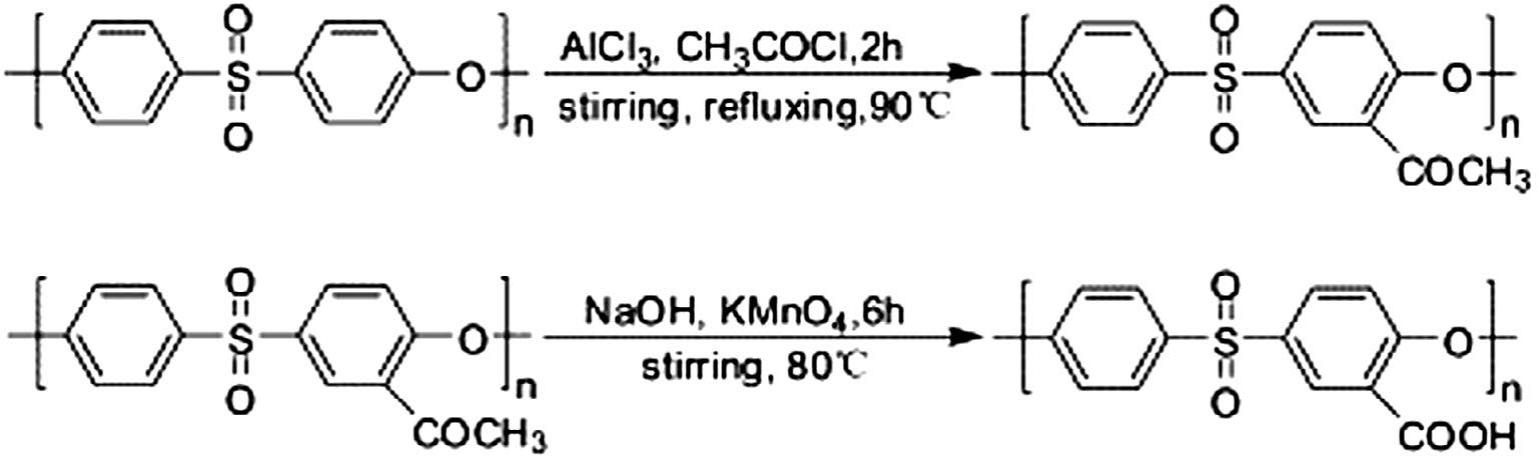
The two step process for the carboxylation of PES [57].

Another method for the carboxylation of PES is the acetylation/ oxidation two steps process, and it is done first by the addition of a mixture of acetyl chloride and AlCl_3_ dissolved in N-methylpyrrolidone (NMP) solution to the PES in NMP solution at 90 °C in order to produce PES–COCH_3_. The second step is the oxidation reaction in which a mixture of KMnO_4_/NaOH /double distilled water (DDW) solution is added slowly to PES–COCH_3_ in NMP solution at 80 °C in order to obtain the deep red carboxylated PES, Figure 6 [57].

#### 2.3.3. Amination (NH_2_)

**Figure 7.**
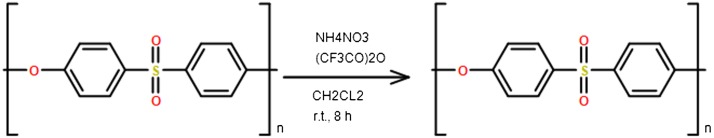
Nitration of PES by two-step process [59].

Bulk modification of PES to attach amine functions is used widely to increase the hydrophilicity of the PES in a similar way to sulfonation and carboxylation. The attachment of the electrophilic amine groups on the polymer chains is very efficient since these groups are very reactive and easily used to covalently bond with so many functional groups [58]. Amino functional groups are mostly attached to PES backbone by the two-step process; a nitration reaction followed by a reduction reaction, but they can be also attached to the polymer chain by polymerization with monomers that contain amino groups.The modification of PES to introduce NH_2_ is usually thought to be difficult, but the reverse is correct. That is because of the presence of strong electron withdrawing groups such as sulfonyl functional groups, which makes phenylene rings are highly electron deficient, so it enhances nucleophilic-substitution reaction on the aryl ether bonds [4]. Hence, introduction of NH_2_ functional groups is used as one of the most critical technique to enhance the antifouling effects of PES membrane. Aminated polyethersulfon (NH_2_-PES) are generated from the reduction of nitro polyethersulfone (NO_2_-PES) which they can be synthesized mostly by HNO_3_, HNO_3_+H_2_SO_4_/oleum mixture, or by using a mixture of ammonium nitrate (AN) and trifluoroacetic anhydride (TFAA) [59] . Botvey et al. [59] has synthesized nitrated PES with DS of 0.80 by suspending the polymer in dichloromethane (DCM) with AN, and TFAA was then added slowly while cooling to the flask and stirred for 8 h. Then, the reaction mixture was participated by dropping the solution in water and filtered by filtration to obtain NO_2_-PES, as illustrated in Figure 7.

**Figure 8.**
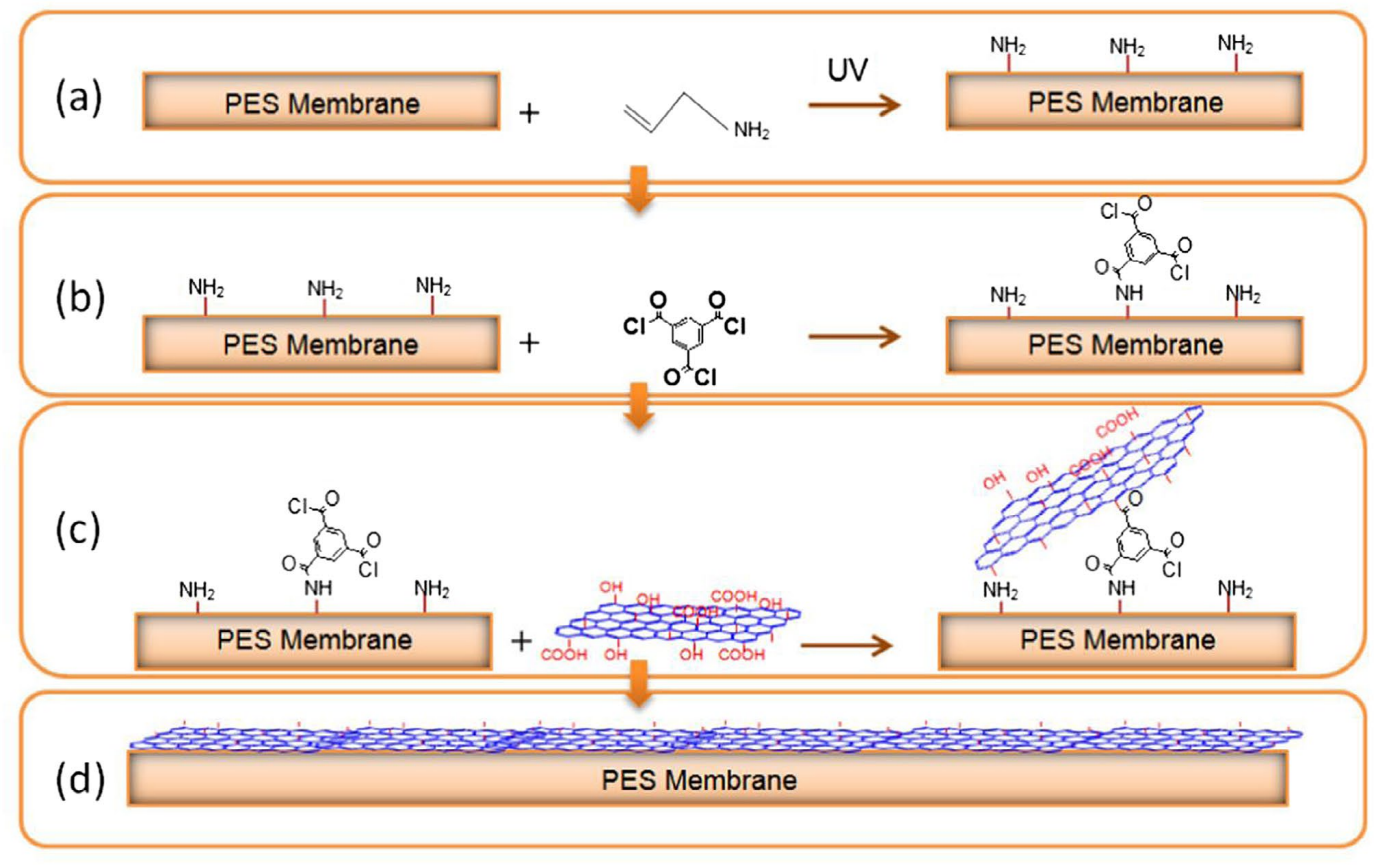
Reaction diagram of the surface modification of PES membrane using GO. (a) PES amination through UV irradiation; (b) Reaction between PES grafted NH_2_ and TMC; (c) Reaction between GO and PES modified surface with TMC; (d) Representation diagram of the modified PES membrane with GO [24].

**Figure 9.**
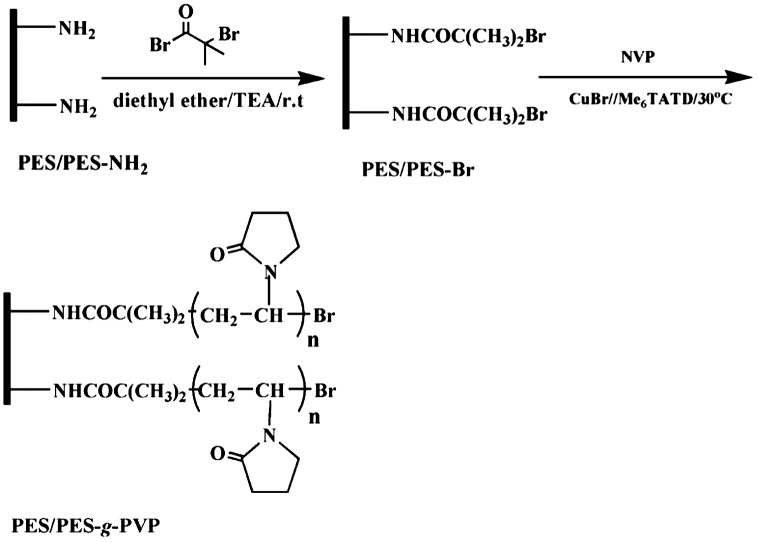
Scheme representation of the preparation of initiator-functionalized PES/PES-Br membrane and PVP-grafted membranes [27].

**Figure 10.**
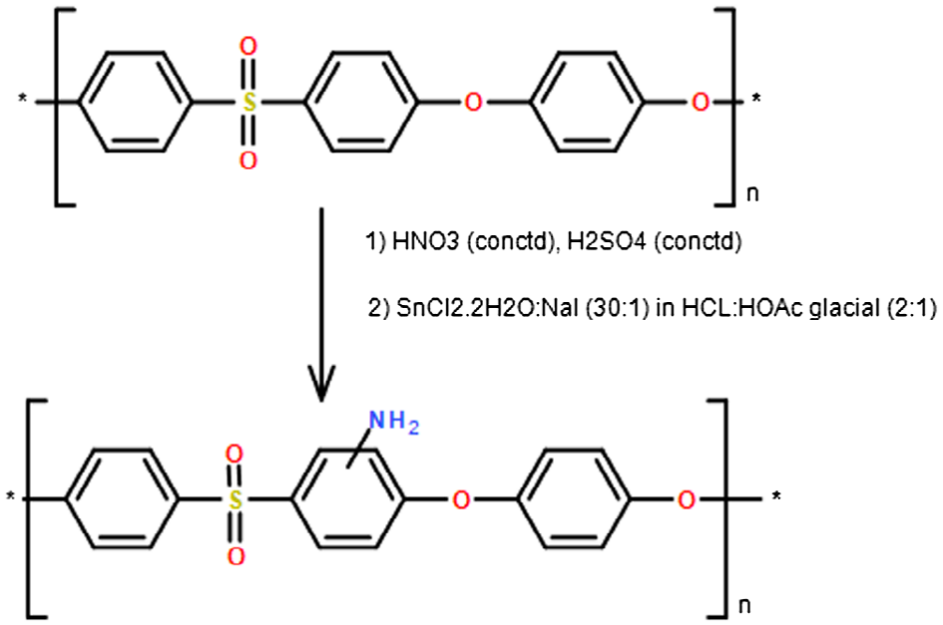
Schematic illustration of PES amination. PES (upper) and NH_2_-PES (lower) [61].

**Figure 11.**
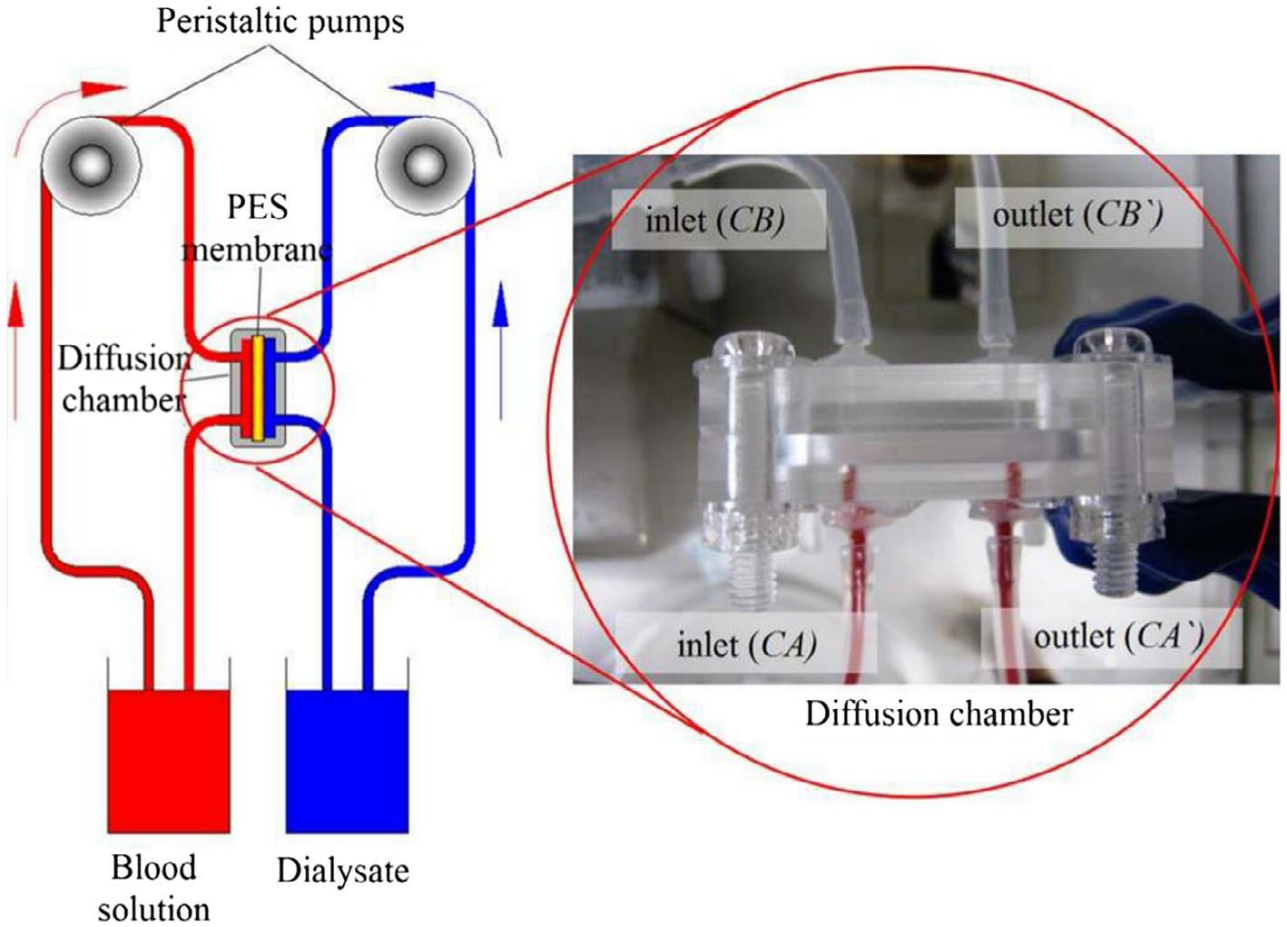
The membranes diffusion test for over 28 days. A loop system and diffusion chamber [66].

**Figure 12.**
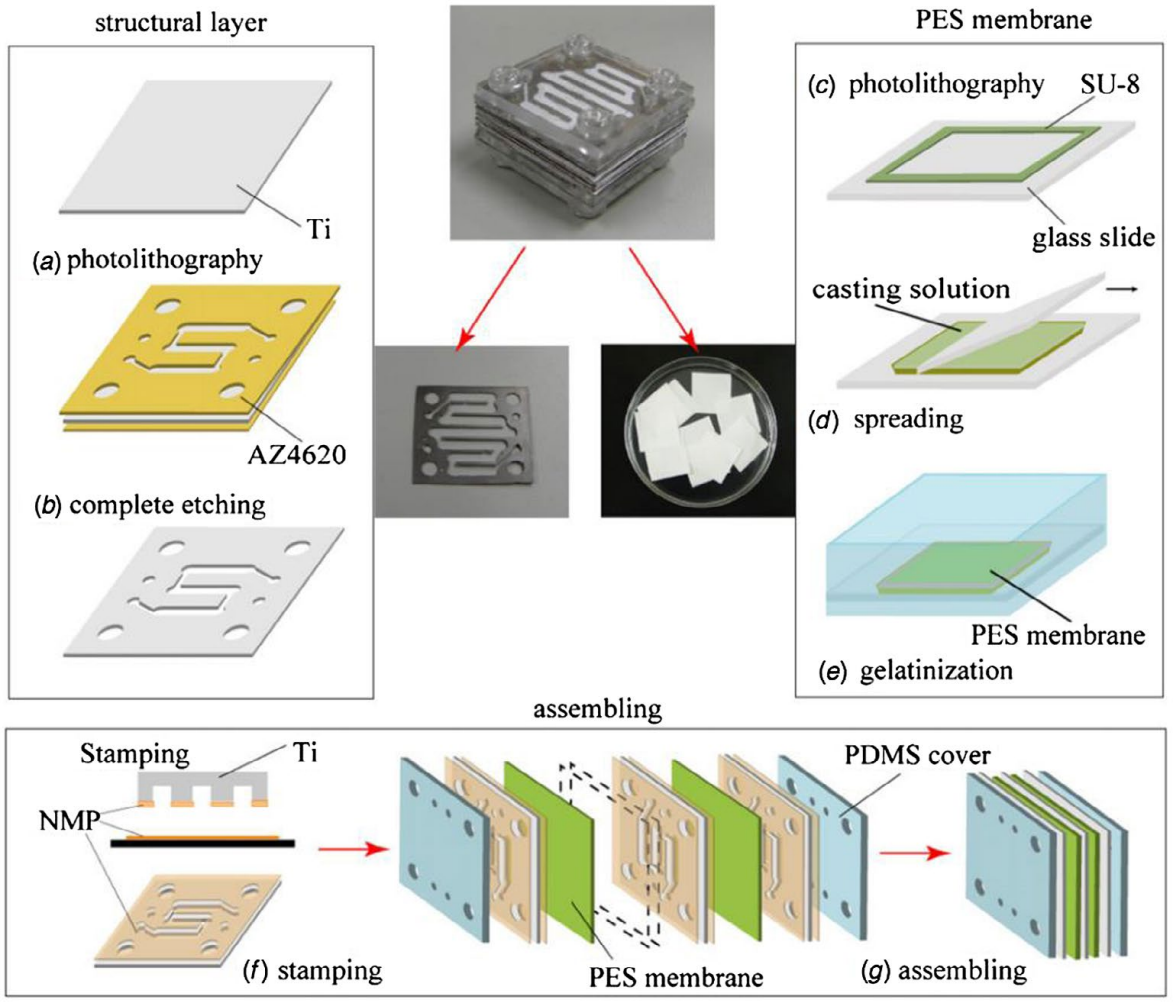
The production process of the multilayered microfilter device [67].

**Scheme 1.**
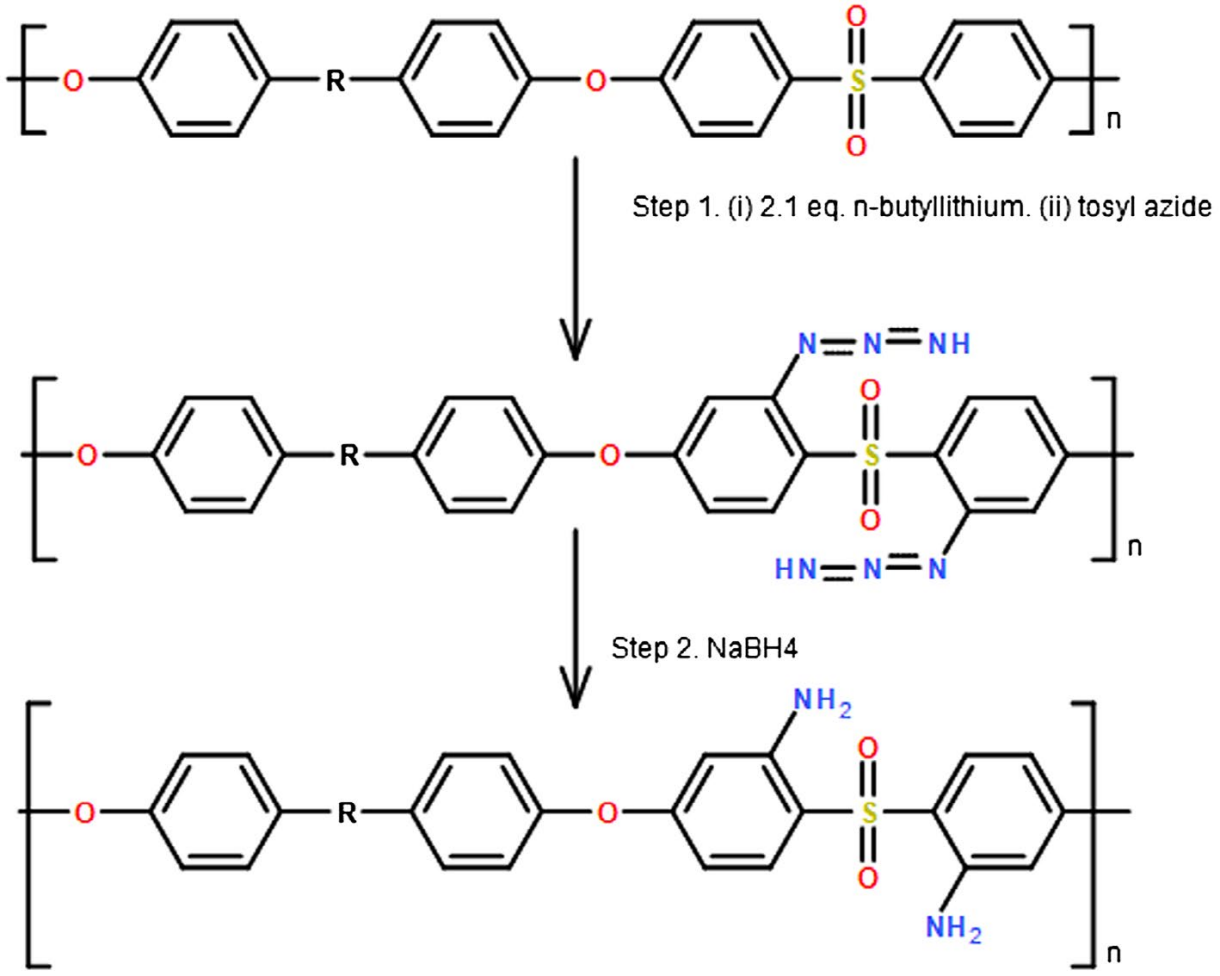
Preparation of ortho sulfone amine **3** from poly(ary1sulfone) **1** through azide intermediates **2** [58].

Guiver et al. [58] has investigated three approaches for amination of polysulfones/poly(ary1sulfone) first by bromine replacement on dibrominated polysulfones with nucleophilic NH_2_ synthons, aromatic ring direct amination using electrophilic NH_2_ synthons, and lithiated polymers amination using azides and other electrophilic NH_2_ synthons. In the first approach, the di-brominated polysulfones was aminated using sodium amide in liquid ammonia by an elimination-addition reaction of halogenobenzene on polymer chains leading to aminobenzenes. The degree of the polymerization (DS) was about 0.33 at −78 °C while at −33 °C, the products were insoluble. Hence, potassium amide was recommended as a reagent instead of sodium amide for better solubility in ammonia. In the second approach, hydroxylamine-O-sulfonicacid (HOSA) is used a reagent for the amination of aromatic rings in DMF using different conditions, but no reaction happened with the polymer. Also, when trimethylsilyl azide is used as a reagent for amination of polysulfones under different conditions in the presence triflic acid and in chloroform as a solvent, the DS was below 0.3, and sever polymer chain degradation occurred due to the use of triflic acid. In third approach, Poly (aryl sulfone (ortho sulfone) diazide or were synthesized by the reaction of butyllithium and polysulfones, then reacted with tosyl azide. The resulted polymeric material was followed by a reduction step in order to give complete conversion of the azide derivatives to poly (ary1sulfone (ortho sulfone) diamine using sodium borohydride as shown in Scheme 1. DS was about 2.0 or less through controlling the butyllithium quantity during the direct lithiation process, and conversion was mono-substituted amine repeated unit. However, when lithiated dibrominated polymeric material was used as a starting material and reacted with 3.1 equiv. of n-butyl lithium in order produce azide derivatives followed by the reduction step with sodium borohydride. The DS increased up to 2.0–2.75 also through controlling the lithiation process, and the conversion was di-substituted amine repeated unit.

## 3. The utilization of NH_2_-PES with nanomaterials/monomers/enzymes

**Scheme 2.**
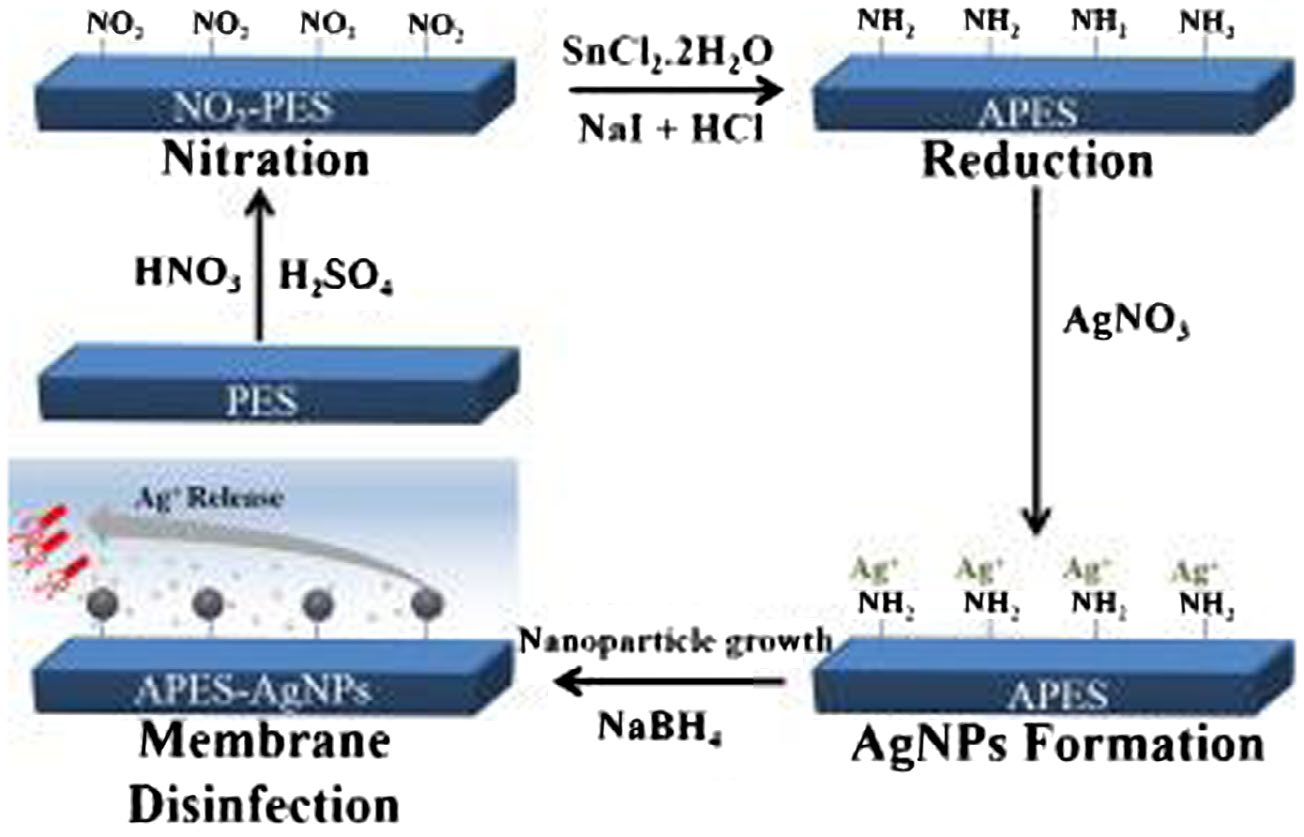
Ag-NH_2_-PES membrane fabrication suggested by Haider et al. [9].

NH_2_-PES is utilized with different nanomaterials to obtain modified NH_2_-PES membrane with better antifouling effects as well as biological effects. In a recent study by Haider et al. [9], NH_2_-PES was fabricated by a nitration reaction with nitric-sulfuric acids followed by a reduction reaction using SnCl_2_·2H_2_O. Then, silver (Ag) nanoparticles were attached to NH_2_-PES membrane either by immobilization surface technique using NaBH_4_ as a reducing agent to attach Ag nanoparticles with NH_2_ groups on the surface, or by blending technique via blending/embedding Ag particles with NH_2_-PES. The immobilized or blended Ag-NH_2_-PES membranes were then tested against *E. coli* bacteria. Membranes with a high antibacterial activity will possess high antifouling properties, which prolonged the life of the water treatment membrane. In general, the results showed the addition of Ag particles to NH_2_-PES provided higher antibacterial activity than the bare NH_2_-PES membrane. Specifically, the attachment of Ag nanoparticles on the surface of NH_2_-PES membrane showed higher antibacterial activity than the embedded/blended Ag particles in NH_2_-PES. This is because that immobilization of Ag nanoparticles on the membrane surface allows a direct interaction between Ag nanoparticles and bacteria while embedding the Ag particles in the polymeric membrane could somewhat hinders this interaction. Hence, the attachment of amino groups on the PES surface is a critical step to immobilize Ag nanoparticles on the surface of the membrane in order to induce antibacterial and prevent antifouling in the water treatment membrane (Scheme 2).

**Scheme 3.**
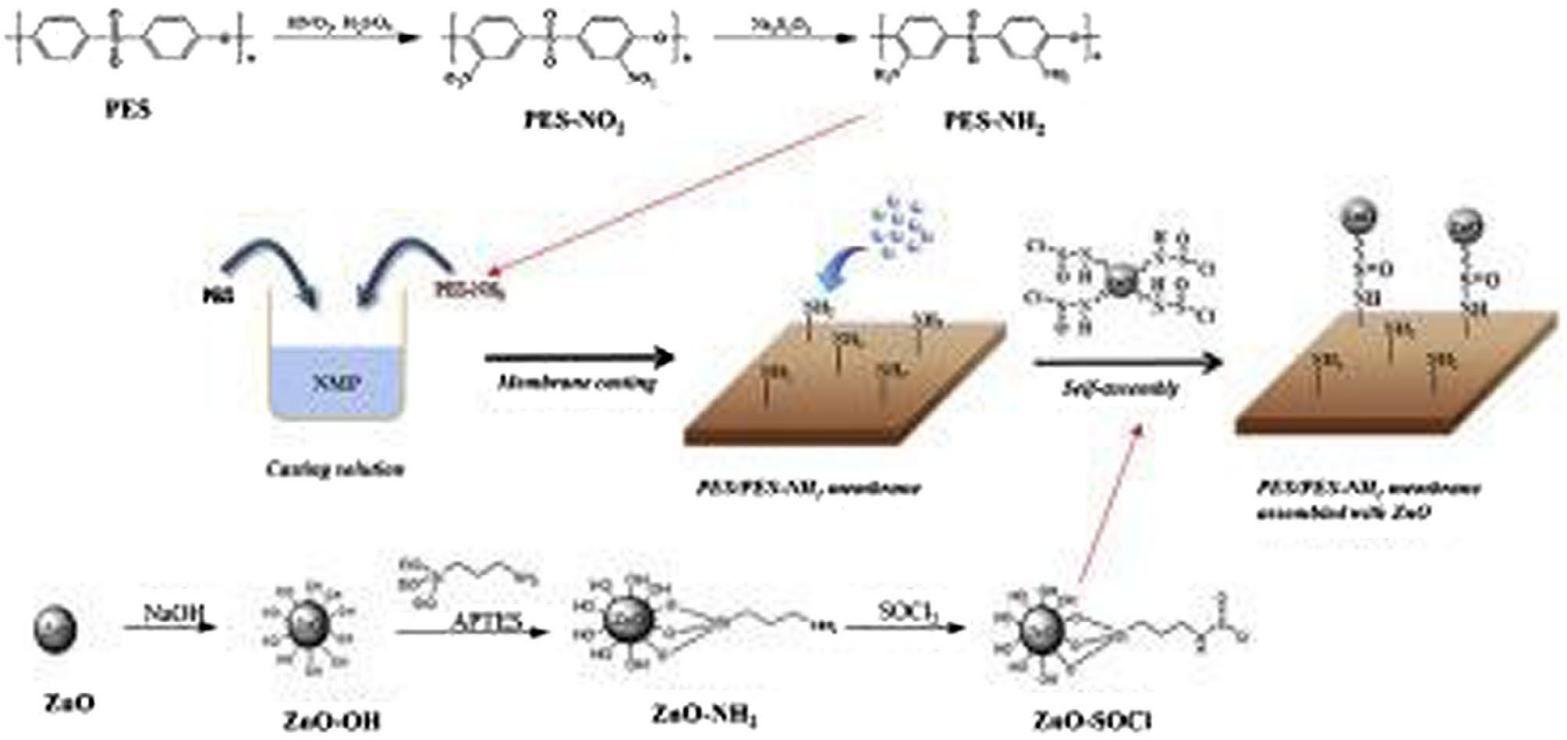
Self-assembled of ZnO particles on NH_2_-PES/PES membrane [60].

Jo et al. [60] have used ZnO nanomaterials with the NH_2_-PES to enhance the antifouling effects of the membrane. In this study, NH_2_-PES was synthesized first by nitration with nitric-sulfuric acids followed by a reduction reaction with Na_2_S_2_O_4_. Then, ZnO nanoparticles were assembled on the surface of NH_2_-PES/PES membrane by reacting thionyl chloride-terminated ZnO nanoparticles with NH_2_ functional groups in NH_2_-PES/PES membrane as shown in Scheme 3. The results of the study showed that ZnO-NH_2_-PES/PES membrane (with at least 0.8 wt.% ZnO nanomaterials) exhibited antibacterial activity against both *E*. *coli* and *S. aureus*, a higher water flux, and a better fouling resistance without a loss in solute rejection in comparison to the bare NH_2_-PES/PES membrane.

In another study by Vatanpour et al. [31], multi walled carbon nanotubes (MWCTs-NH_2_) with concentrations of 0.015, 0.03, 0.045 and 0.06 wt.% were fabricated as nanofilers and blended with PES mix matrix. The MWCTs was prepared first by reacting them with nitric-sulfuric acids then with thionyl chloride (SOCl_3_) to produce MWCTs-COCl, and then reacted with 4,4′-diamino diphenyl methane (DDM) to produce MWCTs-NH_2_. This nanofiler blended in different concentrations with PES to produce MWCTs-NH_2_-PES mix matrix membrane. In this study, amino functional groups used in order to disperse MWCTs and increase functional groups in PES mix matrix, which eventually caused an increase in both the hydrophilicity and water flux of the membrane. The results also showed that the salt rejection increased as NH_2_-MWCNTs content increased. Specifically, NH_2_-MWCNTs with 0.045 wt.% in PES membrane showed the highest pure water flux up to 23.7 L/m^2^ h and showed the best antifouling properties because its test results exhibited lower surface roughness and higher negative surface charge compare with the others concentrations.

Another study utilized the amine functions in PES with graphine oxide (GO) nanoparticles was done by Igbinigun et al. [24] in which GO nanosheets was introduced onto the surface of allylamine-PES membrane in order to develop new antifouling PES membranes. In this study, GO nanosheets were covalently bind to the modified PES membrane by three-step process as shown in Figure 8. In the first step, amine functional groups were grafted on the PES membrane by polymerizing of allylamine on the surface of PES membrane using UV-induced grafting technique. Then, Go nanosheets were covalently attached to the modified allylamine-PES membrane by using a cross-linking reagent, trimesoyl chloride (TMC). The acyl chloride groups of the cross linker present on the surface of PES membranes reacted with hydroxyl and carboxylic functional groups in GO nanosheets to create ester or anhydride linkages. The results of the study showed the modified PES membrane with GO nanosheets had a smooth surface, and the hydrophilicity, negative zeta potential, flux recovery of the modified membrane increased noticeably as well as lower fouling effects in comparison with unmodified membrane.

Xiang and his coworkers [27] used PES/PES-NH_2_ membrane to graft poly (N-vinylpyrrolidone) (PVP) on the surface of the membrane in order to decrease protein adsorption, and eventually the membrane can be used in blood purification applications. The PES-NH_2_ was synthesized in two step processes nitration then followed by reduction using SnCl_2_. 2 wt.% of PES-NH_2_ was used in order to make PVP grafted PES membranes which was prepared using surface-initiated atom transfer radical polymerization (SI-ATRP) as shown in Figure 9. In this technique, the amino functional groups reacted with the reaction with 2-bromoisobutyryl in mixed solvents of TES: diethyl ether at r.t to prepare initiator-functionalized PES membrane. Afterwards, SI-ATRP of NVP was proceeded at 30 °C on the initiator-functionalized membrane. In this the study, CuBr/Me6 TATD was used as a catalyst due to its high reactivity, and deionized distilled water was used a solvent in this reaction. The results of this modification showed the grafting of PVP on the modified PES membrane increased with an increase of the reaction time to reach of 2.3 mg/cm^2^ after 4 h which indicate that SI-ATRP of NVP could be controlled. Also, the results showed the grafted NVP modified membranes exhibited low adsorption to protein such as bovine serum albumin (BSA) and bovine serum fibrinogen (BFG).

In a study by Handayani et al. [61], NH_2_-PES was used as a support material for enzyme immpolization. The NH_2_-PES was synthesized by nitration of PES using nitric-sulfuric acid then followed by a reduction reaction using SnCl_2_·2H_2_O as shown in Figure 10.

In this study, PES/PES–NH_2_ was used as a supporting material to immobilize enzymes in order to be utilized as bioreactors with high lipase performances. The results of the study showed the largest enzyme loading was for *Mucor miehei* lipase that was immobilized on a PES–NH_2_ membrane containing 10% of PES–NH_2_, 8% of dibutyl phthalate (DBP), and 5% of polyethylene glycol (PEG) (872.62 μg/cm^2^). The results of the study also showed that the immobilization of lipases on PES–NH_2_ did not affect their biocatalysts activity, and the reusability test showed when lipases are immobilized on PES–NH_2_, a better constancy and recovery (up to 97.16%) were obtained in comparison with unmodified PES, with only 95.37%.

Furthermore, in biomedical applications, PES membrane has been used in blood purification, biomedical devices, hemodialysis systems, and artificial kidneys [26]. The wide use of PES membrane in dialysis systems is due to its permeability and diffusion properties. However, several research studies are ongoing to enhance blood compatibility of the PES membrane since a low membrane compatibility will eventually affects the permeability when the membrane is applied in blood devices [62–65]. Prihandana et al. [66] have prepared PES membrane coated with Parylene film in order to enhance blood compatibility of the membrane. The results showed after 28 days of the membrane testing (a long-term diffusion testing for PES membrane as illustrated in Figure 11), 90% of unmodified PES membrane was covered with platelets, whereas the biocompatibility of the PES membrane coated with parylene film had enhanced significantly with only 20–30% a platelet coverage. In case of the permeability, the results showed the permeability of the unmodified PES membrane significantly decreased during the first 7 of the membrane testing and became stable after 8 days while the permeability of modified PES membrane coated with parylene film showed a constant performance though out the membrane testing which indicate its potential use in hemodialysis applications.

The diffusivity of the membranes was evaluated over a long term of 28 days. A loop system and diffusion chamber, as illustrated in Figure 8, was designed and fabricated to represent a portable dialysis system for long-term diffusion tests on the membranes. In this system, a peristaltic pump (Peri-Star Pro, World Precision Instruments) was used to circulate the blood and dialysate into the diffusion chambe.

In addition, Gu et al. [67] has prepared a multilayered microfilter in order to be used as a wearable artificial kidney dialyzer to separate metabolic wastes such as urea, uric acid and creatinine from blood. The PES membrane was selected as a separation membrane for the wearable artificial kidney due to its good mechanical strength, thermal stability, and chemical resistance. This microfilter system was made by the assembly of titanium layers and the nano-porous of PES membrane as shown in Figure 12. The results of the study showed that the urea diffusion rate was about 18 μg min^−1^ per layer. In comparison to a human kidney, the kidney size fabricated device showed different flow capacity depending on the velocities of the aqueous solutions. Hence, when the solutions have velocities similar to pure water, the flow capacity was 1220 ml min^−1^, but when the solutions have velocities similar real blood, the flow capacity was about 300–400 ml min^−1^. Nevertheless, the fabricated device showed a 2.5 times urea removal rate than a human kidney which indicates a potential use of PES fabricated dialyzer device in hemodialysis applications.

## 4. Conclusions

Chemical modification of PES membrane including surface, blending, and bulk modifications are critical techniques used to diminish its biofouling effects. Surface modification to introduce hydrophilic functional groups is considered one of the most used option to enhance the hydrophilicity of PES without affecting the mechanical and thermal properties of PES backbone. Amino functions have been effectively employed to enhance the hydrophilicity of PES surface and to introduce monomers, enzymes, and nanomaterials to PES surface, which have been shown to enhance the biological activity of this polymeric material. Most of the previous research studies have synthesized amino functionalized PES membranes by a nitration reaction with H_2_SO_4_:NHO_3_ then followed by a reduction reaction. Modification of PES with amine functions do not only enhance the hydrophilicity of PES membrane, but they also possess potential coordinate bonding sites to different nanomaterial in order to be introduced on the membrane surface. Nanomaterials such as Ag, GO, ZnO, and MWCNs are far as this moment were the only nanoparticles that have been explored previously with NH_2_-PES membrane. Further studies are needed to enhance the immobilization of nanomaterials on the surface of aminated functionalized PES membrane to ensure that the nanomaterials do not leak to the aquatic environments.

## Disclosure statement

No potential conflict of interest was reported by the authors.
